# Dysregulation of calcium homeostasis in cancer and its role in chemoresistance

**DOI:** 10.20517/cdr.2023.145

**Published:** 2024-03-15

**Authors:** Neema Kumari, Narasimha Pullaguri, Subha Narayan Rath, Ashish Bajaj, Vikas Sahu, Kranti Kiran Reddy Ealla

**Affiliations:** ^1^Department of Microbiology, Malla Reddy Institute of Medical Sciences, Hyderabad 500055, India.; ^2^Research & Development division, Hetero Biopharma Limited, Jadcherla 509301, India.; ^3^Department of Biomedical Engineering, Indian Institute of Technology, Hyderabad 502284, India.; ^4^National Reference Laboratory, Oncquest Laboratories Ltd., Gurugram 122001, India.; ^5^Department of Oral and Maxillofacial Pathology, Malla Reddy Institute of Dental Sciences, Hyderabad 500055, India.; ^#^Authors contributed equally.

**Keywords:** Calcium, cancer, chemoresistance, exosome, apoptosis, calcium channels, multidrug resistance

## Abstract

Globally, cancer, as a major public health concern, poses a severe threat to people’s well-being. Advanced and specialized therapies can now cure the majority of people with early-stage cancer. However, emerging resistance to traditional and novel chemotherapeutic drugs remains a serious issue in clinical medicine. Chemoresistance often leads to cancer recurrence, metastasis, and increased mortality, accounting for 90% of chemotherapy failures. Thus, it is important to understand the molecular mechanisms of chemoresistance and find novel therapeutic approaches for cancer treatment. Among the several factors responsible for chemoresistance, calcium (Ca^2+^) dysregulation plays a significant role in cancer progression and chemoresistance. Therefore, targeting this derailed Ca^2+^ signalling for cancer therapy has become an emerging research area. Of note, the Ca^2+^ signal and its proteins are a multifaceted and potent tool by which cells achieve specific outcomes. Depending on cell survival needs, Ca^2+^ is either upregulated or downregulated in both chemosensitive and chemoresistant cancer cells. Consequently, the appropriate treatment should be selected based on Ca^2+^ signalling dysregulation. This review discusses the role of Ca^2+^ in cancer cells and the targeting of Ca^2+^ channels, pumps, and exchangers. Furthermore, we have emphasised the role of Ca^2+^ in chemoresistance and therapeutic strategies. In conclusion, targeting Ca^2+^ signalling is a multifaceted process. Methods such as site-specific drug delivery, target-based drug-designing, and targeting two or more Ca^2+^ proteins simultaneously may be explored; however, further clinical studies are essential to validate Ca^2+^ blockers’ anti-cancer efficacy.

## INTRODUCTION

Despite advances in technology and available treatments, cancer remains the second leading global cause of death^[1]^. With improved therapeutic approaches, most patients with early-stage cancer are now curable. There are several treatment options, including conventional and advanced treatments, based on cancer stage, histology, and biomarkers. Conventional treatments are chemotherapy, radiation, and surgery, while advanced treatment options include hyperthermia, ferroptosis-based therapy, immunotherapy, stem cell transplant, targeted therapy, hormone therapy, gene therapy, phototherapy, and others^[2,3]^. Of these, chemotherapy is the most preferred mode of treatment by oncologists as a primary treatment to reduce the cancer burden. Abraxane, bortezomib, cisplatin, docetaxel, doxorubicin, paclitaxel, and temozolomide are some examples of anti-cancer drugs used in chemotherapy^[4]^. However, chemotherapy has many challenges, such as non-specific drugs, side effects, rapid metabolism of drugs, and ineffective drugs. Furthermore, 90% of the major factor contributing to chemotherapy failure is chemoresistance^[5]^. Intrinsic or acquired chemoresistance is a major hurdle in successful cancer treatment as it often leads to cancer relapse and metastasis, which, in turn, reduces the efficacy of cancer treatment and causes patient death. This resistance can arise either to a single anti-cancer drug or to multiple anti-cancer drugs that differ structurally and/or functionally^[6,7]^. This is due to underlying genetic mutations, tumour heterogeneity, activated intrinsic pathways, pharmacological factors, and the tumour microenvironment (TME)^[3,7,8]^. Moreover, it is vital to find novel therapeutic strategies to overcome chemoresistance. For this, it is necessary to understand the molecular mechanisms of chemoresistance, which include tumour suppressor genes, transporters, pumps, oncogenes, mitochondrial alteration, autophagy, epithelial-mesenchymal transition (EMT), DNA repair, cancer stemness, and exosome^[9-11]^.

Several studies have emphasised the role of calcium (Ca^2+^) in cancer progression and chemoresistance^[12]^. Being a secondary messenger, Ca^2+^ plays a crucial role in maintaining cellular functions, apoptosis, and regulating the network of Ca^2+^ signalling in cells. Disrupted Ca^2+^ signalling networks in cancer cells implicate a critical role of Ca^2+^ in assisting cancer cells in growing and overcoming the anti-cancer effects of drugs. In the past decade, there have been significant breakthroughs in our understanding of how the Ca^2+^ signal may influence or even drive pathways that are crucial for therapeutic resistance in cancer. Since it is not possible to review the large number of studies done in this area, cancer studies targeting the Ca^2+^ signalling proteins are briefly described here. In addition, studies targeting Ca^2+^ signalling and its proteins to overcome chemoresistance are also summarised. Thus, in this review, we aim to understand the role and potential therapeutic strategies of targeting Ca^2+^ proteins in chemoresistant cancer cells.

## Ca^2+^ SIGNALLING VIA Ca^2+^ CHANNELS, PUMPS, AND EXCHANGERS

**Figure 1:**
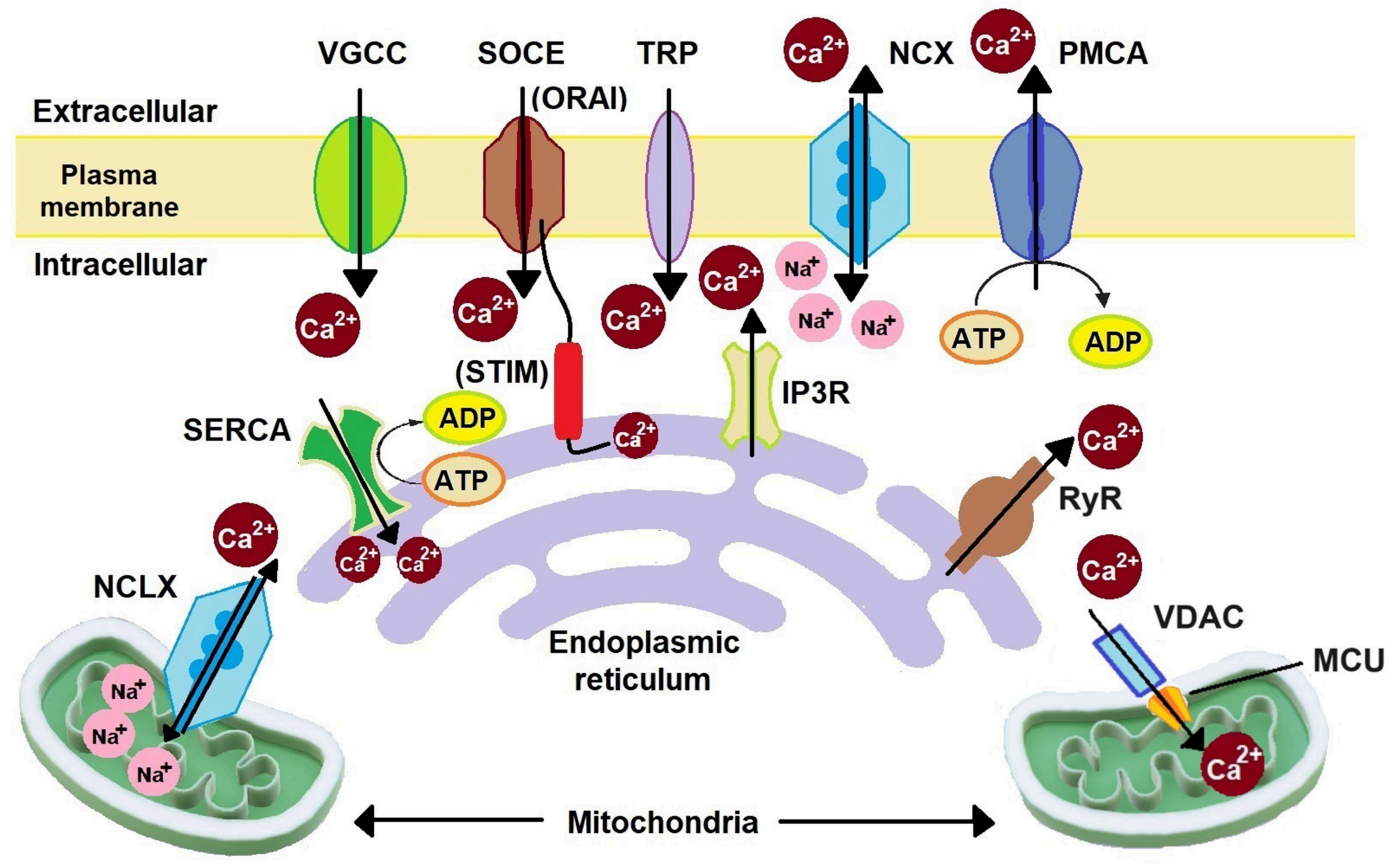
Schematic representation of major Ca^2+^ signalling proteins. Extracellular Ca^2+^ is transported to the cytoplasm through VGCCs, SOCC (ORAI), and TRP channels. Organelle Ca^2+^ is transported to the cytoplasm through mitochondrial NCLX, IP3R, and RyR. Cytoplasmic Ca^2+^ is removed by NCX, PMCA, SOCC (STIM), SERCA, and MCU. NCLX: Na^+^/Ca^2+^/Li^+^ exchanger; SERCA: sarco/endoplasmic reticulum Ca^2+^ ATPase; VGCCs: voltage-gated Ca^2+^ channels; SOCE: store-operated Ca^2+^ entry; STIM: stromal interacting molecule; TRP: transient receptor potential; IP3R: inositol-1,4,5-trisphosphate receptor; NCX: Na^+^/Ca^2+^ exchanger; PMCA: plasma membrane Ca^2+^ ATPase; RyR: ryanodine receptor; VDAC: voltage-dependent anion channel; MCU: mitochondrial Ca^2+^ uniporter; SOCC: store-operated Ca^2+^ channels.

Ca^2+^ is a multifaceted tool that plays a significant role as a secondary messenger in several cellular functions, such as cell growth and division, migration, absorption, secretion, transcellular transport, signalling, and apoptosis^[13]^. In cells in the resting phase, the cytoplasmic Ca^2+^ concentration ([Ca^2+^]_cyt_) is 100 nM and the extracellular Ca^2+^ concentration ([Ca^2+^]_ext_) is 1-2 mM. In intracellular Ca^2+^ stores, such as endoplasmic reticulum (ER) ([Ca^2+^]_ER_), the Ca^2+^ concentration is > 100 µM. In mitochondria ([Ca^2+^]_mit_) and the nucleus, the Ca^2+^ concentration remains the same as in the cytoplasm. Upon activation of the Ca^2+^ signalling proteins, the [Ca^2+^]_cyt_ can exceed 1 µM^[14-18]^. This changed Ca^2+^ level initiates several signalling cascades. Sustained elevation of Ca^2+^ levels can lead to cell death; hence, a controlled Ca^2+^ level is maintained by Ca^2+^ channels, pumps, and exchangers. As illustrated in Figure 1, these Ca^2+^-related proteins are located on both the plasma membrane and cellular organelles, including the ER, mitochondria, Golgi apparatus (GA), and lysosomes^[18-20]^. Ca^2+^ binding proteins present in cells are calmodulin (CaM), parvalbumin, calcineurin, S100 protein, and several other proteins that are present in both extracellular and intracellular regions^[21]^.

On the plasma membrane, voltage-gated Ca^2+^ channels (VGCC), store-operated Ca^2+^ channels (SOCC), transient receptor potential (TRP) channels, Na^+^/Ca^2+^ exchanger (NCX), and plasma membrane Ca^2+^ ATPase (PMCA) are present. Depending on the electrophysiological properties and the genes encoding them, there are five types of VGCC: L-type (LTCC), P/Q type (P/QTCC), N-type (NTCC), R-type (RTCC), and T-type (TTCC). LTCC has four isoforms: Ca_v_1.1-1.4. P/QTCC, NTCC, and RTCC each have one isoform: Ca_v_2.1, Ca_v_2.2, and Ca_v_2.3, respectively. TTCC has three isoforms: Ca_v_3.1-3.2^[22]^. These channels are activated upon reaching their respective voltage threshold during the voltage change in the membrane. LTCC, P/QTCC, NTCC, and RTCC are high-voltage-activated (HVA) channels, while TTCC is a low-voltage-activated (LVA) channel. Therefore, HVA channels are mostly found in excitable cells, and TTCC is found mostly in non-excitable cells. However, HVA channels are also found to be expressed in some non-excitable cells^[18]^. SOCC has two subunits: ORAI and stromal interacting molecule (STIM). ORAI is present on the plasma membrane, while STIM is located on the ER. ORAI has three isoforms: ORAI1-3. STIM has two isoforms: STIM1 and STIM2^[23]^. Upon Ca^2+^ depletion in the ER, ORAI and STIM proteins are relocalized to plasma membrane-ER junctions and interact afterwards with each other to activate Ca^2+^ influx^[18,24-26]^. This process is termed store-operated Ca^2+^ entry (SOCE). ORAI channels can also lead to Ca^2+^ influx driven by arachidonic acid without interacting with STIM proteins^[18]^. TRP has 28 different families divided into six subfamilies encoded by *TRP* genes such as TRPC (canonical), TRPV (vanilloids), TRPM (melastatin), TRPA (ankyrin), TRPP (polycystins), and TRPML (mucolipins)^[27,28]^. TRPC7 enhances cell growth and migration with the activation of Ca^2+^/CaM-dependent protein kinase II (CAMKII), phosphatidylinositol 3-kinase (PI3K)/Akt, and extracellular signal-regulated kinase (ERK)^[29]^. Three isoforms of NCX are found in cells: NCX1-3^[30]^. NCX functions in both forward and reverse modes. In the forward mode, it transfers three Na^+^ ions inside and two Ca^2+^ ions outside the cells and thus acts as an anti-apoptotic channel. In the reverse mode, it transfers three Na^+^ ions outside and two Ca^2+^ ions inside and thus acts as a pro-apoptotic channel by increasing the Ca^2+^ accumulation in the cells^[31]^. PMCA is an ATP-based Ca^2+^ pump, helping to maintain low [Ca^2+^]_cyt_. PMCA has four isoforms: PMCA1-4^[32,33]^. This pump removes one Ca^2+^ ion per ATP molecule utilized^[33]^.

On the ER membrane, sarco/endoplasmic reticulum Ca^2+^-ATPase (SERCA), STIM, inositol-1,4,5-trisphosphate receptor (IP3R), and ryanodine receptor (RyR) are present. Unlike PMCA, SERCA removes two Ca^2+^ ions from the cytosol to the ER^[33]^. Ca^2+^ release from the ER takes place with the help of IP3R and RyR. IP3R has three isoforms: IP3R1-3^[26]^. IP3R is involved in SOCE by reducing [Ca^2+^]_ER_ and thereby stimulating the activation of STIM1 and ORAI1 to replenish the ER Ca^2+^ level^[26]^. RyR has three isoforms: RyR1-3^[34]^. Upon VGCC activation, the influx of Ca^2+^ from the extracellular region increases [Ca^2+^]_cyt_, which results in the inactivation of VGCC in a negative feedback mechanism. The resulting increased Ca^2+^ ions in the cytosol then induce activation of RyR, which in turn releases Ca^2+^ from the ER, and thus the process is called “Ca^2+^-induced-Ca^2+^-release” (CICR)^[34-36]^. On mitochondria, the mitochondrial Ca^2+^ uniporter (MCU) complex and Na^+^/Ca^2+^/Li^+^ exchanger (NCLX) are present. Cytosolic Ca^2+^ enters mitochondria through the MCU complex, controlled by a voltage-dependent anion channel (VDAC)^[37]^. Like NCX, NCLX also functions in forward and reverse modes. In the forward mode, NCLX transfers Ca^2+^ from mitochondria to the cytosol, and in the reverse mode, it transfers Ca^2+^ from the cytosol to mitochondria^[38]^. In the GA, along with IP3R, RyR, and SERCA, the secretory-pathway Ca^2+^ ATPase (SPCA) is also expressed. SPCA has two isoforms, SPCA1 and SPCA2. It transfers one Ca^2+^ ion at a time inside GA^[39]^. Lysosomes are also known to function as Ca^2+^ stores, though less extensively than the ER. Endocytosis, Ca^2+^/H^+^ exchanger, TRP channels, and multiple Ca^2+^ sensors including CaM help in Ca^2+^ homeostasis in lysosomes^[18,20]^.

During the cell cycle, Ca^2+^ is required in the early G1 phase and in the transition from G1 to S, G2 to M, and metaphase to anaphase. CaM and its downstream targets, CaMKII, calcineurin, and protein kinase C (PKC), are direct effectors of Ca^2+^ signalling. In turn, they regulate transcription factors such as the nuclear factor of activated T cells (NFATs), cyclic adenosine monophosphate (cAMP)-responsive element-binding (CREB) protein, and nuclear factor-κB (NFκB) for cell-cycle progression^[12,13,40]^. CaM assists in the progression through the G1 and M phases. Calcineurin is required for the transition from the G1 to S phases and cyclin D1 expression during the G1 phase^[12]^. Ca^2+^ and CaMKII also regulate centrosome duplication and the separation of chromosomes into daughter cells^[12]^.

### Ca^2+^ signalling in cancer cells

In pathological conditions like cancer, Ca^2+^ signalling is frequently disrupted due to the dysregulation of various Ca^2+^ channels, pumps, exchangers, and binding/storage proteins^[13,41]^. This dysregulation is caused by mutations, changes in expression, regulation, and/or subcellular targeting of the Ca^2+^ signalling proteins^[13]^. Excessive Ca^2+^ levels are toxic to cells, thus requiring tight regulation of Ca^2+^ signalling. Despite this, cancer cells can thrive in high-Ca^2+^ environments due to their additional need for Ca^2+^ to support increased cell division, cell signalling, and tissue invasion^[18]^. In contrast to this, some studies have reported that cancer cells have a reduced dependence on Ca^2+^ for proliferation^[12]^ and evading apoptosis^[13]^.

Henceforth, dysregulated Ca^2+^ signalling proteins either increase or decrease Ca^2+^ levels in cancer cells depending on their perceived benefit to survival. Reduced expression of some VGCCs in cancer tissue compared to normal tissue is also seen, indicating the possible role of those Ca^2+^ channels as tumour suppressor gene markers^[42]^. Studies have also shown that monomeric and heteromeric channels exhibit different advantages or disadvantages to cancer cells. For example, ORAI1-ORAI3 heteromers induce apoptosis resistance in prostate cancer cells compared to ORAI1 monomers through reduced SOCE because the number of ORAI1 monomers is reduced. Similarly, because of the increase in the number of this heteromer, there is an increased proliferation signalling via arachidonic acid^[40,43]^.

In cancer cells, increased production of reactive oxygen species (ROS) leads to the accumulation of ROS at the plasma membrane. This leads to post-translational modification of the plasma membrane channels, thereby promoting Ca^2+^ entry into the cells to counteract the ROS activities^[18]^. Furthermore, disrupted Ca^2+^ signalling promotes the activation of Ca^2+^-dependent transcription factors such as NFAT and G1 cyclins such as c-Myc, c-Jun, and c-Fos. These factors promote cancer growth by inducing the expression of G1 and G1/S phase transition cyclins and associated cyclin-dependent kinases (CDK4 and CDK2)^[12,13]^. Ca^2+^ is also involved in pathways such as mitogen-activated protein kinases (MAPK) and PI3K/Akt that promote malignancy in cancer cells^[29]^.

**Figure 2:**
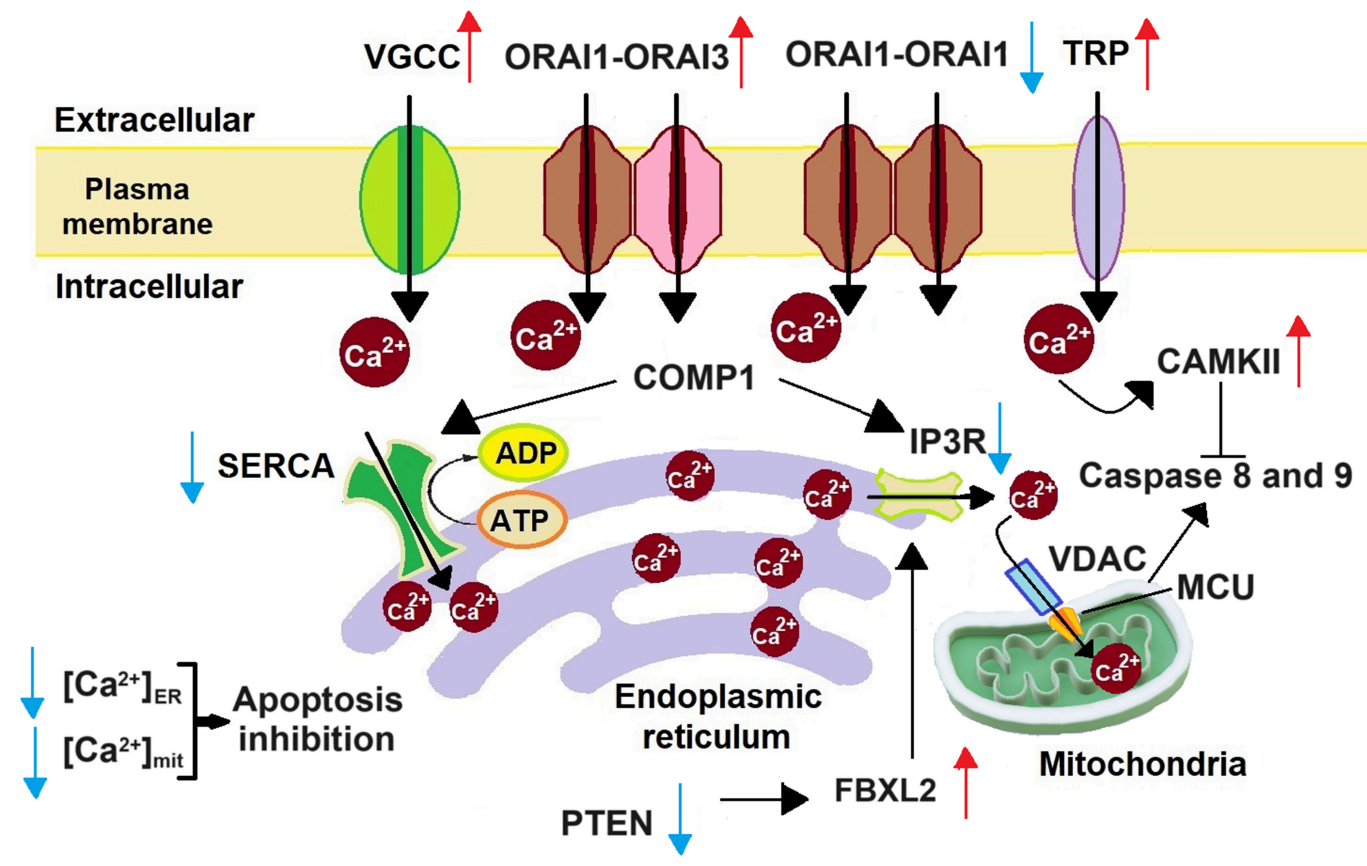
Apoptosis inhibition in cancer cells. Increased Ca^2+^ influx through upregulated VGCC, TRP, and ORAI1-ORAI3 heterodimer leads to activation of CAMKII, which inhibits pro-apoptotic caspases 8 and 9. Downregulation of ORAI1 monomer and SERCA reduces [Ca^2+^]_ER_. IP3R downregulation leads to decreased [Ca^2+^]_mit_ and hence inhibition of caspases 8 and 9. Reduced [Ca^2+^]_ER_ and [Ca^2+^]_mit_ lead to apoptosis inhibition. (red up arrow: upregulation, blue down arrow: downregulation, 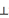: inhibition). SERCA: sarco/endoplasmic reticulum Ca^2+^ ATPase; VGCC: voltage-gated Ca^2+^ channel; TRP: transient receptor potential; COMP1: cartilage oligomeric matrix protein; IP3R: inositol-1,4,5-trisphosphate receptor; VDAC: voltage-dependent anion channel; CAMKII: Ca^2+^/CaM-dependent protein kinase II; MCU: mitochondrial Ca^2+^ uniporter; PTEN: phosphatase and tensin homolog; FBXL2: F-box protein.

Excessive Ca^2+^ stimulates Ca^2+^-sensitive catabolic enzymes such as proteases and endonucleases^[44]^. As shown in Figure 2, elevated [Ca^2+^]_cyt_, facilitated by channels such as TRP and ORAI1-ORA3 heteromers, activates CaMKII, ultimately inhibiting caspases 8 and 9 and promoting cell survival^[43]^. However, cancer cells show reduced Ca^2+^ signalling from the ER required for apoptosis. Therefore, blockage of SERCA by Cartilage Oligomeric Matrix Protein (COMP1) reduces Ca^2+^ uptake in the ER. Furthermore, ORAI1 monomers are responsible for ER-induced apoptosis. Thus, downregulation of ORAI1 further inhibits apoptosis^[43]^. B-cell lymphoma-2 (Bcl-2), an anti-apoptotic protein, modulates ER Ca^2+^ signalling to regulate the apoptosis pathway. Several studies have shown that Bcl-2 is overexpressed in cancer cells^[45]^. Oncogenes such as rat sarcoma (*RAS*), *Akt*, and *miR-25* also regulate Ca^2+^ signalling or proteins responsible for Ca^2+^-dependent apoptosis to prevent apoptosis^[46]^. Increased [Ca^2+^]_mit_ is also associated with apoptosis. Increased Ca^2+^ levels in mitochondria reduce its membrane integrity by opening the mitochondrial permeability transition pore. Subsequently, pro-apoptotic factors are released from mitochondria^[40]^. However, regulation of oncogenes such as Kristen rat sarcoma (*KRAS*) 2 viral oncogene homolog and tumour suppressor proteins such as p53 by Ca^2+^ signalling subsequently reduces [Ca^2+^]_mit_ and thereby promotes cancer cell survival^[25,40]^. Mitochondrial-induced apoptosis is also inhibited by downregulating IP3R. IP3R is downregulated by COMP1 and F-box protein (FBXL2). Phosphatase and tensin homolog (PTEN) (deleted on chromosome 10) inhibits the activity of FBXL2. However, its mutation or inactivation leads to the upregulation of FBXL2, which targets IP3R3 for proteasome degradation^[17,43]^. IP3R downregulation reduces [Ca^2+^]_mit_ and thereby prevents mitochondrial-induced apoptosis. Apart from apoptosis, Ca^2+^ also regulates other cell death modes such as autophagy, necrosis, anoikis, entosis, and ferroptosis, reviewed in other papers^[40,44,47,48]^. Although there is still a lack of clear understanding regarding how Ca^2+^ regulates different cell death modes, increased and sustained [Ca^2+^]_cyt_ is often associated with necrosis and other death modes such as necroptosis^[40]^.

Ca^2+^ signalling is known to regulate many immune-associated pathways in cancer cells^[27]^. Examples include the activation of STAT3, a transcription factor in cancer, as well as MAPK pathway, Akt pathway, and Wnt pathway^[27]^. TME is also remodelled due to Ca^2+^ signalling^[27]^. Ca^2+^ also induces chemoresistance by facilitating cancer cell invasion and metastasis^[25]^. Metastasis accounts for more than 90% of cancer-related deaths. Overexpression of several Ca^2+^ proteins is associated with metastasis^[25]^. Prerequisite steps in metastasis such as pro-migratory signals, movement rate, directional control, and extracellular matrix (ECM) degradation are dependent on Ca^2+^ signalling^[40]^. Channels like ORAI1 and TRPV2 play roles in cancer cell invasion through the ECM and formation of invadopodia. Other pathways such as ROS production and cAMP pathways are also modulated by Ca^2+^, contributing to cancer cell migration and invasion. However, Ca^2+^ proteins involved in cancer cell migration and invasion vary among cancer types and stimuli^[40]^.

Furthermore, Ca^2+^ signalling is also known to regulate epigenetic mechanisms^[49]^. Changes in the DNA methylation and histone post-translational modifications are in good relation with the altered gene expression of Ca^2+^ signalling proteins such as the cadherin and VGCCs in all breast cancer subtypes^[50]^. Targeting Ca^2+^ signalling can reactivate tumour suppressor genes silenced by cancer cell epigenetics. In colon cancer, altered Ca^2+^ signalling results in activated Ca^2+^ CaM kinase which plays a central role in tumour-suppressor genes reactivation and suppressing cancer cell growth^[51]^. Program death ligand 1 (PDL-1), an immune suppressive molecule that binds to receptor program death protein 1 (PD-1) and promotes tumour progression by evading immune response, is present in exosomes produced from cancer cells. Ca^2+^ signalling is known to control both the generation and secretion of exosomes produced from cancer cells^[52]^ by influencing the Rab GTPase family and membrane fusion factors^[53]^. Preclinical studies with CT26 tumour-bearing mice have mentioned that blocking Ca^2+^ channels using dimethyl amiloride (DMA) inhibits exosome release^[54]^. Combining anti-PDL-1 antibody therapy with exosome secretion inhibition using Ca^2+^ channel blockers (CCBs) may enhance efficacy. Exosome PDL-1 represents a prominent therapeutic target in immunotherapy resistance^[52]^.

## TARGETING Ca^2+^ SIGNALLING PROTEINS INVOLVED IN CANCER

### Ca^2+^ proteins present in the plasma membrane

The plasma membrane serves as a crucial interface for Ca^2+^ signalling. Targeting the plasma membrane Ca^2+^ signalling proteins can significantly impact [Ca^2+^]_cyt_ by modulating the entry and exit of Ca^2+^ ions through VGCCs, ORAI, TRPs, NCX, and PMCA. This modulation can either decrease or increase [Ca^2+^]_cyt_, thereby placing a burden on intracellular Ca^2+^ signalling proteins to maintain [Ca^2+^]_cyt_. However, sustained modulation is impractical due to the interconnected activities of plasma membrane and intracellular proteins.

Dysregulation of various channels, pumps, transporters, and proteins occurs in different cancer types, leading to either upregulation or downregulation depending on their survival benefits^[42,55]^. For instance, in a clinical study on lung adenocarcinoma, TRPC7 expression varied among patients^[29]^. Consequently, studies have explored the modulation of Ca^2+^ levels as a therapeutic target. In hepatocarcinoma cells, fluorouracil (5FU) induced cell death by inhibiting Ca^2+^ influx through ORAI1 channels^[56]^, while in colon carcinoma cells, it exerted cytotoxic effects by elevating [Ca^2+^]_cyt_ level to activate CaM, triggering apoptosis via p53 phosphorylation^[57]^.

Overexpression of Ca^2+^ channels, pumps, or exchangers is associated with cancer prognosis. For example, NTCC overexpression correlates with poor survival in patients with non-small cell lung cancer (NSCLC)^[58]^ and adult adrenocortical carcinoma (ACC)^[59]^, while it predicts good prognosis in gliomas^[60]^. Similarly, TRPM2 expression negatively correlates with prognosis in hepatocellular carcinoma patients^[61]^. In lung adenocarcinoma patients, overexpression of TRPC7 is associated with poor prognosis and lower survival rates^[29]^. The study by Wang *et al.* has shown that overexpression of PMCA4 is associated with good prognosis in gastric cancer patients^[62]^.

Targeting these proteins induces cancer cell death through various modes, including apoptosis, autophagy, necrosis, entosis, and ferroptosis. Different channels are associated with different modes of cell death. For instance, ORAI1 plays a role in entosis in cancer development via the septin-ORAI1-Ca^2+^/CaM-myosin light chain kinase (MLCK)-actomyosin axis^[63]^. The study by Lee and Park showed that when blocking or knocking out the ORAI1 channel, there was reduced entosis in the MCF7 breast cancer cells^[63]^. Notably, the role of entosis in cancer remains unclear^[64]^, but both invading and engulfing cells require intracellular Ca^2+^ signalling for entosis.

### Ca^2+^ proteins present in intracellular organelles

Similar to plasma membrane Ca^2+^ signalling proteins, Ca^2+^ signalling proteins within intracellular organelles are associated with different modes of cell death and cancer prognosis. Reduced [Ca^2+^]_ER_ protects cancer cells from apoptosis and resistance to cisplatin and taxol^[58,65]^; increased [Ca^2+^]_ER_, facilitated by upregulated IP3R and RyR, promotes proliferation, migration, invasion, malignancy, and apoptosis in various cancer cells^[66]^.

Increased [Ca^2+^]_mit_ is associated with apoptosis and necrosis^[58,65]^. MCU promotes ferroptosis under cystine deprivation, while inhibiting ferroptosis induced by glutathione peroxidase 4 (GPX4) inhibition^[67,68]^. Accumulation of lipid ROS, either due to cysteine deprivation or inactivated GPX4 (the enzyme required to remove lipid ROS), leads to ferroptosis^[68]^. Cells overexpressing MCU upregulate cysteine consumption to remove lipid ROS. This leads to cysteine deprivation, which in turn induces the transcription of “Ferroptosis Signature” genes^[67]^. This ferroptosis due to cysteine deprivation is inhibited on MCU knockout^[67]^. In contrast, in a study by Marmolejo-Garza *et al*., it is shown that MICU1 from the MCU complex has a protective role against ferroptosis. On knocking out MICU1 in HT22 (mouse hippocampal neuronal cell line) and mouse embryonic fibroblasts (MEF) cells, they showed increased sensitivity of these cells toward ferroptosis^[69]^. Moreover, altered expression of the MCU complex is associated with poor prognosis, increased metastasis, migration, invasion, and metabolic stress resistance in cancers^[67,70]^.

Furthermore, SPCA1 and SPCA2 overexpression in the GA is associated with a pro-survival role in breast cancer^[71-73]^. Knocking down SPCA1^[73]^ and SPCA2^[72]^ has shown to reduce MDA-MB-231^[73]^ and MCF-7^[72]^ breast cancer cells proliferation, respectively. Knockdown of SPCA2 has also shown anchorage-independent growth of MCF-7 cells and tumour formation in mice models^[72,73]^. However, the role of SPCA2 in tumour progression is indirect by increasing Ca^2+^ influx across the plasma membrane by activating ORAI1 channels after SPCA2 is localized at the plasma membrane on its overexpression^[73]^.

Further, it is reported that lysosomal Ca^2+^ signalling through TRPML1 and TPC2 regulates autophagy^[20,74]^. Targeting these channels has been shown to impair autophagy in non-cancer cells such as ARPE-19 (retinal pigment epithelium cell line)^[74]^, thereby indicating the possibility of deregulating autophagy in cancer cells by targeting TRPM channels. In addition to autophagy, lysosomal Ca^2+^ signalling is also involved in proliferation, metastasis, angiogenesis, and multidrug resistance^[74]^.

Table 1 summarizes the channels, pumps, and exchangers targeted in various studies and their effects on the subjects. Data reveal that each protein exhibits cell-specific, isoform-specific, and treatment-specific effects.

**Table 1:** List of channels, pumps, and exchangers targeted in various studies and the effects on the targeted subjects

**Ca^2+^proteins**	**Isoforms**		
LTCC	Not defined^[75]^, Ca_v_1.2^[76]^	Subjects	Colon cancer cell lines (AZ-97 and HT-29)^[75],^ ameloblastoma primary cells and cell line (AM-1)^[76]^, mice^[76]^
		Treatment	BayK 8644^[75,76]^, Verapamil^[75,76]^, siRNA^[76]^
		Effects	Apoptosis^BayK8644[75]^, apoptosis inhibition^Verapamil[75]^, suppressed cell aggregation and collective migration^[76]^, reduced invasion^[76]^
P/QTCC	Ca_v_2.1	Subjects	Neuroblastoma cells (SH-SY5Y)^[77]^
		Treatment	Ca_v_2.1 mutants^[77]^
		Effects	Reduced proliferation^[77]^, apoptosis^[77]^, decreased Bcl-2/Bax ratio^[77]^
NTCC	Ca_v_2.2	Subjects	Neuroblastoma glioma cells (NG108-15)^[78]^
		Treatment	Cannabinoid^[78]^
		Effects	Inhibition of Ca^2+^ current^[78]^
RTCC	Ca_v_2.3	Subjects	Neuroendocrine tumour BON cells^[79]^
		Treatment	SNX-482^[79]^
		Effects	Decreased chromogranin A secretion^[79]^, reduced resting [Ca^2+^]_cyt_^[79]^
TTCC	Ca_v_3.1^[80]^, Ca_v_3.2^[80,81]^, Ca_v_3.3^[80]^, not defined^[82-85]^	Subjects	Breast cancer cell line (T-47D)^[80]^, glioblastoma cell lines^[81]^, mice^[82]^, lung cancer cell line (A549)^[83,84]^, leukaemia cell lines^[86]^
		Treatment	NNC-55-0396^[80,82,86]^, mibefradil^[81,86]^, RNA interference^[81,82]^, KYS05090^[83]^, TTA-A2^[84,85]^
		Effects	Reduced proliferation^[80-82,84,86]^, apoptosis^[81,83,85,86]^, autophagy^[83]^, increased sensitivity to anti-cancer drug^[81,82]^, no effect on tumour size^[82]^, inhibition of survivin^[81]^, activation of BAX, Caspase 9, PARP, p27, and Rb^[81]^, cell cycle arrest^[81]^ at G1 phase^[82,86]^, inhibition of stemness marker CD133^[81]^, increased expression of astrocytic marker GFAP^[81]^, increased ROS level^[83]^, reduced viability^[84]^, reduced wound healing^[84]^, altered cell and spheroid morphology^[84,85]^, reduced colony formation efficiency^[85]^
SOCC	ORAI1^[63,87,88]^, ORAI3^[89,90]^	Subjects	Breast cancer cell line (MCF-7)^[63,89]^, prostate cancer cell line^[90]^, NSCLC cells^[88]^, thyroid cancer cell line^[87]^
		Treatment	RNA interference^[89,90]^, AnCoA4^[63]^, knockdown^[63,87]^, Afatinib^[88]^
		Effects	Apoptosis^[89,90]^, autophagy^[88]^, reduced proliferation^[87,89,90]^, cell cycle arrest at G1 phase^[87,89]^/G2-M phase^[90]^, altered cell cycle proteins^[89]^, reduced entosis^[63]^, reduced cell viability^[88]^, reduced invasion^[87]^
	STIM1^[87,88]^	Subjects	Non-small cell lung cancer cells^[88]^, thyroid cancer cells^[87]^, zebrafish^[87]^, patient samples^[87]^
		Treatment	Afatinib^[88]^, knockdown^[87]^
		Effects	Autophagy^[88]^, reduced cell viability^[88]^, reduced invasion^[87]^, reduced proliferation^[87]^, increased sensitivity to anti-cancer drug^[87]^, reduced tumour growth^[87]^, apoptosis^[87]^, cell cycle arrest at G1 phase^[87]^
TRP	TRPC7^[29]^, TRPM2^[61,91]^	Subjects	Patient-derived lung adenocarcinoma tissue^[29]^, lung adenocarcinoma cells^[29]^, lung squamous cell carcinoma cells^[29]^, HCC cell lines^[61]^, patient-derived HCC tissue^[61]^, human adrenal gland and neuroblastoma tissues^[91]^, neuroblastoma cell line (SH-SY5Y)^[91]^, athymic female mice^[91]^
		Treatment	siRNA^[29]^, A10^[61]^, N-(p-amylcinnamoyl) ACA^[61]^, lentivirus-coated shRNA^[61]^, clotrimazole^[91]^, TRPM2-S (short length of TRPM2)^[91]^
		Effects	Reduced proliferation^[29,61]^, reduced cell migration^[29]^, reduced invasion^[29]^, altered cell cycle proteins^[61]^, slowed growth of patient-derived xenografts and Huh-7 xenografts in mice^[61]^, cell cycle arrest at G0/G1^[29]^ or G1/S phase^[61]^, increased sensitivity to anti-cancer drug^[91]^, reduced tumour volume^[91]^, reduced autophagy/mitophagy^[91]^
NCX	Not defined^[30]^	Subjects	Glioblastoma cell line (U251)^[30]^
		Treatment	Nickel^[30]^, bepridil^[30]^
		Effects	Reduced wound healing^[30]^, reduced proliferation^[30]^
PMCA	Not defined^[92]^, PMCA2^[93]^, PMCA1^[32,62]^, PMCA4^[32,62]^	Subjects	Prostate cancer cell line (PC-3)^[92]^, breast cancer cell line (MDA-MB 231)^[32,93]^, gastric cancer cell lines^[62]^, mice^[62]^
		Treatment	trans-resveratrol^[92]^, siRNA^[32,62,93]^
		Effects	Apoptosis^[92]^, increased [Ca^2+^]_cyt_^[92]^, decreased cell viability^[92]^, cell death in the presence of a stimulus^[93]^, increased sensitivity to anti-cancer drug^[93]^ PMCA1: no effect on cell viability^[32]^, high ionomycin (3 µM) induced necrosis^[32]^, no effect on ABT-263-induced apoptosis^[32]^, no effect on E-cadherin and vimentin expressions^[62]^, no effect on wound healing rate^[62]^ PMCA4: no effect on cell viability^[32]^, no effect on ionomycin (3 µM) induced necrosis^[32]^, augmented ABT-263-induced apoptosis^[32]^, inhibition of NFκB translocation^[32]^, increased metastasis^[62]^, reduced expression of E-cadherin, GRHL2, and OVOL1^[62]^, increased expression of vimentin^[62]^, increased wound healing^[62]^, increased [Ca^2+^]_cyt_^[62]^, increased nuclear NFATc1 accumulation^[62]^
IP3R	Not defined^[94]^	Subjects	T-ALL^[94,95]^, breast adenocarcinoma cell lines^[95]^
		Treatment	Xestospongin B^[94]^, 2APB^[95]^
		Effects	Reduced mitochondrial respiration^[94],^ cell death^[94]^, increased NAD^+^/NADH ratio^[94]^, inhibition of ER-Ca^2+^ release^[95]^, inhibition of ROS production^[95]^, inhibition of immunepotent CRP-induced death^[95]^
SERCA	SERCA2^[88]^	Subjects	NSCLC cells^[88]^
		Treatment	Afatinib^[88]^
		Effects	Autophagy^[88]^, reduced cell viability^[88]^
RyR	Not defined^[95]^, RyR2^[96]^	Subjects	Breast adenocarcinoma cell lines^[95]^, T-ALL cells^[95]^, insulinoma cell line (INS-1)^[96]^
		Treatment	Dantrolene^[95]^, knockout^[96]^
		Effects	Inhibition of ER-Ca^2+^ release^[95]^, inhibition of ROS production^[95]^, inhibition of immunepotent CRP-induced death^[95]^, impaired SOCE^[96]^, reduced [Ca^2+^]_cyt_^[96]^, elevated VGCC current density^[96]^, increased action potential frequency^[96]^
MCU	Not defined^[37,67,70]^	Subjects	MCU-knockout transformed fibroblast cells^[37]^, mice^[37]^, Pancreatic ductal adenocarcinoma tissue^[67]^, mice^[67]^, CRC^[70]^
		Treatment	Knockout^[37,67]^, knockdown^[70]^
		Effects	Small tumour size^[37]^, reduced proliferation^[37,70]^, cell cycle arrest at S phase^[37]^, increased glycolysis^[37]^, inhibited cell migration^[67]^, inhibited invasion^[37,67]^, inhibited soft agar colony formation^[67]^, reduced tumour weight^[67]^, reduced ROS level^[67]^, ferroptosis inhibition^[67]^, Nrf2 upregulation^[67]^, decreased mitochondrial content^[70]^, decreased mtDNA copy number^[70]^, decreased ATP production^[70]^, decreased expression levels of oxidative phosphorylation-related proteins^[70]^
NCLX	Not defined^[67,97]^	Subjects	Pancreatic ductal adenocarcinoma tissue^[67]^, colorectal cancer cell lines^[97]^, knockout mice^[97]^
		Treatment	CGP37157^[67]^, si/shRNA^[97]^
		Effects	Increased ferroptosis and lipid peroxidation in the absence of cysteine^[67]^, no effect on cell viability in presence of cystine^[67]^, 50% less tumours^[97]^, smaller tumour^[97]^, reduced dysplasia^[97]^, increased metastasis^[97]^, reduced proliferation^[97]^, apoptosis^[97]^, increased invasion^[97]^, mitochondrial perturbations^[97]^, increased mitochondrial ROS^[97]^, stem cell-like phenotype^[97]^, chemoresistance of CRC cells^[97]^
SPCA	SPCA2^[71,72]^	Subjects	Breast cancer cell lines^[71,72]^, mice^[72]^
		Treatment	Knockdown^[71,72]^
		Effects	Reduced cell growth^[71,72]^, cell cycle arrest at G0/G1 phase^[71]^, diminished SICE^[71]^, DNA damage^[71]^, increased sensitivity to anti-cancer therapy^[71]^, increased ROS level^[71]^, delayed tumour formation^[72]^

LTCC: L-type Ca^2+^ channel; P/QTCC: P/Q type Ca^2+^ channel; Bcl-2: B-cell lymphoma-2; NTCC: N-type Ca^2+^ channel; RTCC: R-type Ca^2+^ channel; BAX: Bcl-2 associated X-protein; PARP: poly(ADP-ribose) polymerase; GFAP: glial fibrillary acidic protein; ROS: reactive oxygen species; NSCLC: non-small cell lung cancer; SOCC: store-operated Ca^2+^ channels; STIM: stromal interacting molecule; HCC: hepatocellular carcinoma; TRP: transient receptor potential; TRPC7: transient receptor potential canonical 7; TRPM2: transient receptor potential melastatin 2; ACA: anthranilic acid; NCX: Na^+^/Ca^2+^ exchanger; PMCA: plasma membrane Ca^2+^ ATPase; ABT-263: a Bcl-2 family inhibitor; NFκB: nuclear factor-κB; GRHL2: grainyhead like transcription factor 2; OVOL1: ovo like transcriptional repressor 1; NFATc1: nuclear factor of activated T cells 1; T-ALL: T-cell acute lymphoblastic leukaemia; IP3R: inositol-1,4,5-trisphosphate receptor; 2APB: 2-Aminoethoxydiphenyl borate; NAD^+^/NADH: nicotinamide adenine dinucleotide/nicotinamide adenine dinucleotide (NAD) + hydrogen (H); ER: endoplasmic reticulum; CRP: C-reactive protein; SERCA: sarco/endoplasmic reticulum Ca^2+^-ATPase; RyR: ryanodine receptor; SOCE: store-operated Ca^2+^ entry; VGCC: voltage-gated Ca^2+^ channel; MCU: mitochondrial Ca^2+^ uniporter; NCLX: Na^+^/Ca^2+^/Li^+^ exchanger; CRC: colorectal cancer cell lines; SPCA: secretory-pathway Ca^2+^ ATPase; SICE: store-independent Ca^2+^ entry.

## INTERSECTION BETWEEN Ca^2+^ SIGNALLING AND CHEMORESISTANCE: FROM PATHWAYS TO THERAPEUTIC INTERVENTIONS

Chemotherapy often produces encouraging responses in cancer patients. However, patient life expectancy is ultimately constrained by chemotherapy resistance throughout treatment. Ca^2+^ signalling also significantly contributes to chemoresistance in cancer cells. Of note, [Ca^2+^]_cyt_ may play a role in the development of intrinsic and acquired chemoresistance^[98,99]^.

The Ca^2+^ channel TRPC5 has been shown to induce resistance in non-resistant breast cancer cells by increasing Ca^2+^ influx. This typically occurs after the transfer of TRPC5, present in extracellular vesicles of resistant breast cancer cells, to non-resistant breast cancer cells. Subsequently, the Ca^2+^ influx induces the expression of multidrug resistant ATPase 1 (MDR ATPase) via the Ca^2+^-dependent transcription factor, NFATc3. Increased MDR ATPase then removes several types of anti-cancer agents from the cells, leading to chemoresistance^[17,100,101]^.

The complexity of Ca^2+^ signalling and the multitude of Ca^2+^ signalling proteins make overcoming chemoresistance a multifaceted process. Therefore, various approaches can be employed to address intrinsic or prevent acquired chemoresistance. Apart from the possible role of Ca^2+^ pharmacological modulators as standalone anti-cancer drugs, several studies have demonstrated their role as adjuvants in enhancing the anticarcinogenic effects of anti-cancer drugs. However, considering the vast number of research in this field, encompassing all studies is beyond the scope of this review. Consequently, this section briefly discusses the role of Ca^2+^ in the TME, cellular pathways, cancer stem cells (CSCs), utilization of combination and sequential treatments, and relevant clinical studies.

### Ca^2+^ signalling in the TME

The TME is one of the crucial elements in cancer progression, where various cell types, including endothelial cells, mesenchymal cells, immune cells, adipocytes, and pericytes, coexist alongside cancer cells^[102]^. These diverse cell populations help cancer cells by providing mechanical support and releasing cytokines. Tumour development is mediated by anti- or proinflammatory cytokines that shape the TME^[28]^. These changes in the TME pose challenges to successful treatment and help cancer cells evade immune responses^[102]^. Ca^2+^ plays a critical role in supporting cancer cell migration and invasion by phosphorylating contractile proteins, inducing matrix metalloproteinases (MMPs), and remodelling peripheral and focal adhesions^[17]^.

Disrupted Ca^2+^ signalling regulates immune responses, contributing to chemoresistance in cancer cells^[103,104]^. Increased ROS in mitochondria, triggered by Ca^2+^ signalling, phosphorylates and translocates STAT3, a transcription factor, into the nucleus. There, STAT3 activates genes related to immunosuppression^[27]^. Inflammation induced by these mechanisms reduces the efficacy of anti-cancer drugs such as cisplatin^[41]^. Therefore, targeting Ca^2+^ signalling to reduce inflammatory mediators represents a therapeutic approach in cancer treatment. Alhamad *et al*. demonstrated that although BTP2 (a SOCE inhibitor) treatment alone showed no effect on inflammatory mediators when administered in combination with cisplatin, it reversed cisplatin’s effect on increasing the gene expression of inflammatory mediators such as COX2, IL-8, and Tumour Necrosis Factor-α (TNF-α)^[41]^.

Hypoxic conditions within tumours induce EMT, allowing cancer cells to transition from an epithelial to a mesenchymal phenotype, facilitating migration to other locations within the body. Additionally, angiogenesis, another hallmark of cancer, promotes tumour growth and invasiveness^[105]^. Both EMT and angiogenesis are regulated by Ca^2+^ channels such as VGCC and TRP channels. Consequently, chelating cytosolic Ca^2+^ results in the blockage of several EMT markers such as vimentin, Twist, and N-cadherin, potentially inhibiting angiogenesis^[17,27,106]^.

### Ca^2+^ signalling pathways in chemoresistance

Chemoresistance also arises from the activation of various cell signalling pathways including MAPK, CaMKKβ/AMPKα/mTOR, PI3K/Akt, and Wnt/β-catenin pathways^[17,27]^. These pathways are important in diverse cancer cell processes such as tumourigenesis, proliferation, protein synthesis, metastasis, and invasion^[27]^. G-protein coupled receptors (GPCRs) play a central role in translating extracellular signals and regulating Ca^2+^ signalling by coupling to G-proteins, adenylyl cyclase (AC), cAMPs, and protein kinases. Given that GPCRs represent the largest family of membrane proteins and serve as targets for approximately 50% of therapeutic drugs, designing drugs based on GPCRs is crucial^[107]^.

**Figure 3:**
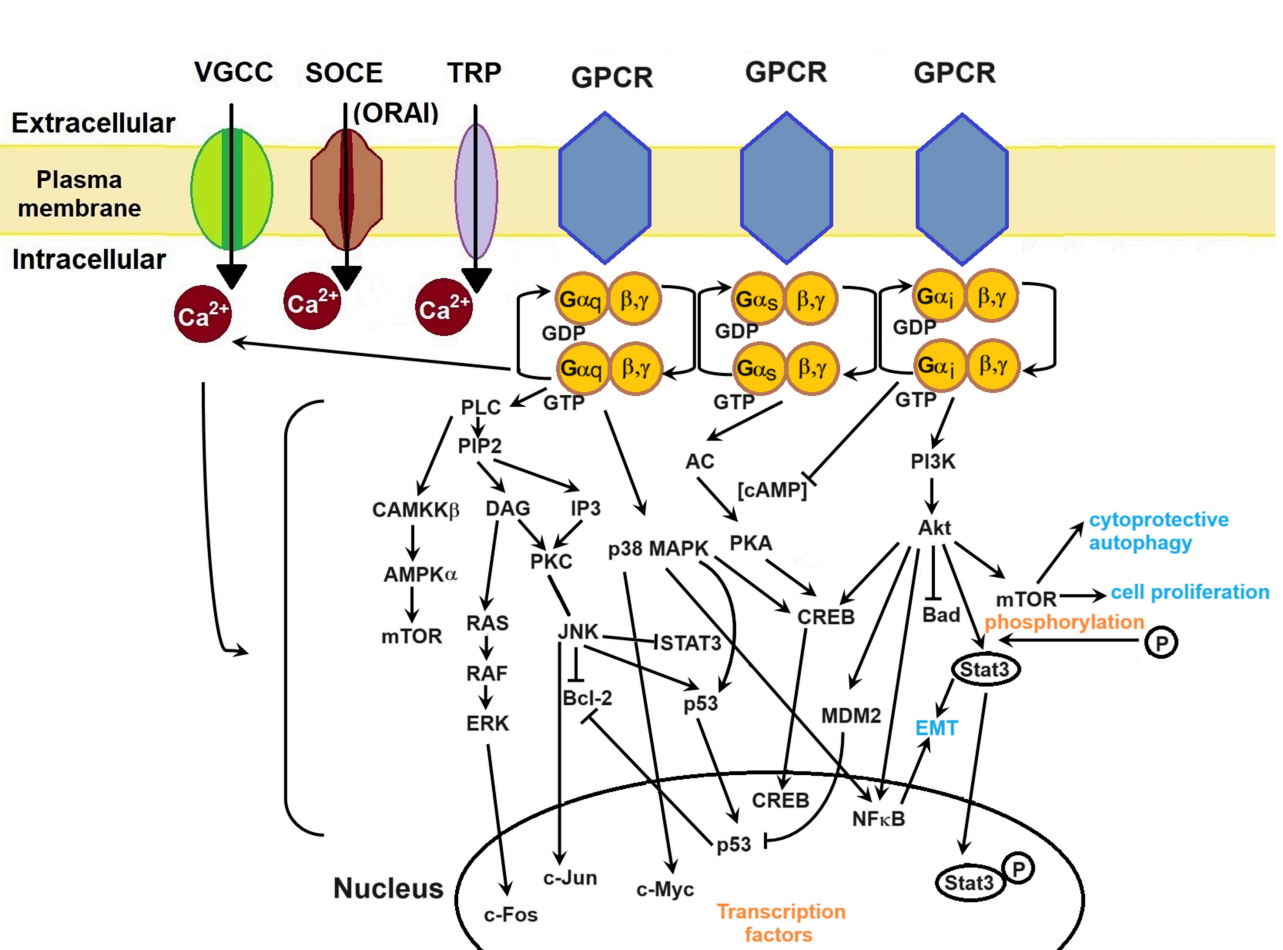
Ca^2+^ signalling pathways in cancer cells. GPCRs mediate cellular pathways such as MAPK, CaMKKβ/AMPKα/mTOR, and PI3K/Akt, thereby exerting regulatory control over Ca^2+^ channels. Consequently, Ca^2+^ ions modulate these cell pathways. The generation and regulation of transcription factors lead to cancer cell proliferation, EMT, metastasis, invasion, and survival. Akt and Murine Double Minute 2 (MDM2) inhibit Bad and p53, respectively, thereby supporting evasion from apoptosis. Inhibition of p53 releases Bcl-2, which in turn inhibits apoptosis. STAT3 and NFκB promote EMT, while mTOR promotes cell proliferation and cytoprotective autophagy. Transcription factors such as c-Fos, c-Jun, c-Myc, and CREB play pivotal roles in gene transcription crucial for cellular growth. VGCC: voltage-gated Ca^2+^ channel; SOCE: store-operated Ca^2+^ entry; TRP: transient receptor potential; GPCR: G-protein coupled receptor; PLC: phospholipase C; PIP2: phosphatidylinositol 4,5-bisphosphate; DAG: diacylglycerol; IP3: inositol-1,4,5-trisphosphate; PKC: protein kinase C; RAS: rat sarcoma; RAF: rapidly accelerated fibrosarcoma; ERK: extracellular signal-regulated kinase; JNK: c-Jun N-terminal kinase; STAT3: signal transducer and activator of transcription 3; MAPK: mitogen-activated protein kinases; NFκB: nuclear factor-κB; CREB: cyclic adenosine monophosphate-responsive element-binding; AC: adenylyl cyclase; PKA: protein kinase A; cAMP: cyclic adenosine monophosphate; PI3K: phosphatidylinositol 3-kinase; EMT: epithelial-mesenchymal transition.

Various G-proteins modulate Ca^2+^ signalling^[108]^, initiating a cascade of intracellular events. While the complexity of cellular pathways involves numerous molecules and signalling cues, providing a detailed description of each pathway is beyond the scope of this review. In brief, GPCRs have three types of G-proteins: G_α_, G_β_, and G_γ_, with G_α_ further subdivided into G_αq_, G_αs_, and G_αi_, each having its unique signal transduction mechanism [Figure 3]. Upon G_αq_ activation, several pathways are initiated, including CaMKKβ/AMPKα/mTOR, and MAPK. The G_αq_ subunit activates phospholipase C (PLC), which catalyzes the conversion of Phosphatidylinositol 4,5-bisphosphate (PIP2) into inositol-1,4,5-trisphosphate (IP3) and diacylglycerol (DAG). IP3 binds to IP3R, initiating the release of Ca^2+^ from the ER into the cytoplasm^[108]^.

Activation of G_αs_ leads to AC activation, initiating cAMP production. cAMP activates protein kinase A (PKA), which phosphorylates proteins regulating excitation-contraction coupling through VGCCs, RyRs, and myosin-binding protein C^[108]^. Upon G_αi_ activation, the G_αi_ subunit inhibits cAMP production and VGCCs^[108]^ while activating the PI3K/Akt pathway. PI3K/Akt promotes cytoprotective autophagy, cell proliferation, EMT, and apoptosis inhibition through the degradation of several molecules such as mTOR, STAT3, NFκB, and p53. For instance, in prostate cancer cells (PCa cells), Ca^2+^ influx through TRPM8 leads to Ca^2+^/CaM activation of Akt kinase, thereby increasing cell proliferation^[43]^.

In a study by Wang *et al.*, it was shown that in the human ovarian cancer cell line A2780 (resistant to paclitaxel), human lung adenocarcinoma cell line A549 (resistant to paclitaxel), human breast cancer cell line MCF7 (resistant to paclitaxel and cisplatin), and human immortalized myelogenous leukaemia cell line K562 (resistant to paclitaxel, cisplatin, and Adriamycin), [Ca^2+^]_cyt_ was reduced. Successful results were obtained in reversing the chemoresistance by chelating Ca^2+^ ions using Ca^2+^-chelated self-assembled nanoparticles (CSNPs). This intervention altered the expression of a chemoresistant gene and induced apoptosis by inhibiting the NFκB signalling pathway^[6]^. TRPC7 has been implicated in mediating CaMKII, Akt, and MAPK signalling, which are crucial in cancer progression. Knockdown or inhibition of TRPC7 has been shown to suppress the activation of CaMKII, Akt, and ERK^[29]^.

### Targeting Ca^2+^ machinery in CSCs

CSCs are robust chemoresistant cells present in a dormant state in various cancer types^[109-111]^. Upon activation and subsequent differentiation into cancer cells, CSCs contribute significantly to cancer relapse. Similar to cancer cells, CSCs also use abnormally activated cell pathways, including Notch, Hedgehog, Wnt/β-catenin, NFκB, PI3K/Akt, and PTEN pathways^[112]^. Therefore, targeting both CSCs and cancer cells would ensure effective cancer treatment and reduce cancer relapse. Studies suggest that Ca^2+^ channels play crucial roles in CSC functioning, with dysregulated Ca^2+^ signalling proteins promoting their differentiation into cancer cells and enhancing chemoresistance^[113,114]^. Dysregulation of various Ca^2+^ channels, including VGCCs^[81,115]^, RyRs^[116,117]^, IP3R^[118]^, SOCE^[119]^, or TRPs^[120,121]^, has been observed in CSCs across different cancer types. Targeting these dysregulated Ca^2+^ channels induces terminal differentiation of CSCs and sensitizes them to chemotherapy.

LTCCs are associated with stem cell differentiation, while TTCCs play a role in cell cycle progression and the self-renewal capacity of undifferentiated stem cells. Additionally, IP3Rs are implicated in maintaining CSC properties such as self-renewal, proliferation, and differentiation^[113]^. Some studies have shown that targeting Ca^2+^ channels inhibits proliferation and induces apoptosis in CSCs. For example, Zhang *et al*. demonstrated that blocking TTCC with mibefradil inhibited the proliferation, survival, and stemness of glioblastoma stem-like cells (GSCs) and sensitized them to temozolomide chemotherapy. This effect was attributed to the inhibition of the pro-survival Akt/mTOR pathway and stimulation of the pro-apoptotic Survivin and Bax pathways. Moreover, TTCC inhibition reduced the expression of oncogenes and increased the expression of tumour suppressor genes^[81]^. In another study, CCBs (manidipine, lacidipine, benidipine, and lomerizine) inhibited stemness and PI3K/Akt and ERK pathways in ovarian CSCs^[112]^.

### Therapeutic interventions targeting Ca^2+^ signalling

Combination therapy utilizing both anti-cancer drugs and Ca^2+^ protein blockers is often studied. Anti-cancer drugs change Ca^2+^ signalling within cancer cells, by targeting pathways related to cell growth or inducing cell death, compromising cellular integrity, reducing ATP production, or blocking specific growth factors^[17]^. The effect of anti-cancer agents on Ca^2+^ signalling depends on the exposure time. During shorter exposure, for up to 8 h, there is an increase in Ca^2+^ influx, ER Ca^2+^ release, and ER-mitochondria Ca^2+^ transfer; however, longer exposure, spanning from hours to weeks, leads to heightened Ca^2+^ efflux, altered ER Ca^2+^ regulation, and elevated activity of the ER Ca^2+^ release channel^[17]^. For example, short-term exposure to 5FU increases Ca^2+^ influx via plasma membrane channels, while long-term exposure increases Ca^2+^ efflux via PMCA^[17]^. Conversely, inhibiting Ca^2+^ channels results in sensitized or increased anti-cancer effects of some drugs by lowering [Ca^2+^]_cyt_. For example, Andrographis sensitizes cancer cells synergistically to gemcitabine^[122]^, cisplatin^[123]^, 5-FU^[124]^, and doxorubicin^[125]^ *in vitro*. Co-treatment of Andrographis and gemcitabine decreases [Ca^2+^]_cyt_ in gemcitabine-resistant cells^[122]^. Co-treatment of cisplatin and SOCE inhibitors increases cisplatin cytotoxicity in cisplatin-resistant breast cancer cells^[41]^ and NSCLC^[126]^. Furthermore, BTP2 treatment reverses cisplatin-induced upregulation of SOCE components (ORAI1 and STIM1) genes^[41]^. Knockdown of ORAI1 and STIM1 has also been shown to sensitize cancer cells to anti-cancer drugs^[87,126,127]^. However, in one study, it was shown that cisplatin cytotoxicity was reduced upon knockdown of ORAI1 and STIM1 in NSCLC and prostate cancer cells^[128,129]^.

Silencing PMCA2 in MDA-MB-231 cells promoted the anti-cancer effects of doxorubicin^[130]^. Blocking TTCC with mibefradil sensitized platinum-resistant ovarian tumours to carboplatin in a mouse model^[131]^ and increased the sensitivity of melanoma cells to MAPK inhibitors^[132]^. In diffuse large B-cell lymphoma (DLBCL) patients resistant to rituximab, co-treatment of rituximab with Bay K8644 (an LTCC agonist) significantly induced apoptosis in DLBCL cell lines and markedly reduced tumour volume and weight in DLBCL patient-derived xenografted mice^[133]^.

This increased sensitization to anti-cancer drugs through co-treatment with CCBs is further enhanced by sequential treatment. Blocking or knocking down Ca^2+^ channels arrest cells at specific phases of the cell cycle. For example, blocking or knockdown of TTCCs can arrest cancer cells at the G0/G1 phase^[134]^. Similarly, in one study, TRPC7 knockdown arrested lung adenocarcinoma cells, H1299, at the G0/G1 phase^[29]^, synchronizing cells to a single phase. Consequently, when an anti-cancer drug such as Taxol, which inhibits microtubule depolymerization and stops cell division^[135]^, is administered, a large number of cells are killed. Another advantage of sequential treatment is the requirement for smaller drug doses compared to single or combination treatments. This was evidenced in one of our studies with A549 3D spheroids^[136]^. Sequential treatment of A549 3D spheroids with a TTCC blocker (TTA-A2) and paclitaxel led to a significant reduction in spheroid viability compared to combination treatment. Sequential treatment not only synchronizes cells to a single phase but also prevents or reduces nonspecific interactions of the second drug. For example, paclitaxel, in addition to microtubules, can also interact with IP3R^[137]^ and other Ca^2+^ channels such as TTCC^[136]^. Consequently, in combination treatment, paclitaxel may compete for binding sites with the TTCC blocker on TTCC. Since the paclitaxel interaction is not as strong as that of specific TTCC blockers, this results in weaker inhibition of these channels by paclitaxel and reduced anti-cancer effects^[136]^.

### Clinical trials with Ca^2+^ protein blockers

The effects of targeting Ca^2+^ proteins in humans are relatively unexplored, as indicated by the limited number of clinical studies^[138-142]^. In a clinical trial by Fu *et al.*, TRPV6 was targeted in individuals with advanced epithelial-origin tumours, revealing that SOR-C13, an inhibitor of TRPV6, exhibit anti-cancer effects without any typical chemotherapy-related complications^[138]^. This study highlights the potential of targeted therapy in managing cancer, and mitigating adverse effects associated with traditional chemotherapy.

Carboxyamido-triazole (CAI), a synthetic inhibitor of non-VGCCs, has been studied in clinical phase IB for recurrent and newly diagnosed glioblastoma and anaplastic gliomas^[141]^, as well as phase II trials for glioblastoma^[139]^ and ovarian cancer^[142]^. In phase IB trials, CAI combined with temozolomide or chemoradiation, showed promising results in terms of safety and efficacy in challenging cases^[141]^. However, in a phase II trial by Mikkelsen *et al*., CAI was tested along with radiation on patients with newly diagnosed glioblastoma multiforme. Results showed that even though CAI was safe to administer, it was ineffective in improving the survival rate^[139]^. In patients with relapsed ovarian cancer, CAI exhibited safety with limited toxicity and stabilized the cancer for over six months^[142]^.

Additionally, a phase I study by Holdhoff *et al*. demonstrated the safety of sequential treatment involving mibefradil followed by temozolomide in glioblastoma patients, with some patients showing partial to complete responses to the regimen^[140]^.

These varying results emphasize the complexity of targeting Ca^2+^ proteins for effective cancer treatments. While inhibitors such as SOR-C13 and CAI exhibit promising results in certain cancer types, their efficacy and impact on survival rates vary across cancer stages and types. Positive results from *in vitro* and *in vivo* studies, coupled with the lack of adequate clinical data, indicate the need for more extensive clinical studies to elucidate the role of targeting Ca^2+^ proteins as an effective cancer treatment option.

## CONCLUSION

Although many novel drugs exhibit promising efficacy against chemoresistant cancers, Ca^2+^ signalling-driven chemoresistance remains a major obstacle. The ubiquitous and varying role of Ca^2+^ in normal and cancer cells complicates targeting approaches and also raises safety concerns. A deeper understanding of Ca^2+^ disruptions across various cancers will facilitate the development of effective therapeutic approaches. Researchers worldwide are exploring innovative strategies, including site-specific drug delivery that can target cancer cells without harming normal cells by taking advantage of cancer biomarkers and designing drugs for cancer-specific Ca^2+^ protein splice variants or their proteins expressed in cancer cells. Simultaneously targeting of multiple dysregulated proteins and exploring intracellular organelles interactions, such as lysosome-mitochondria, using advanced models like 3D cell cultures or patient-derived organoids, are promising approaches. Furthermore, an extensive increase in clinical studies is required to establish the anti-cancer efficacy of Ca^2+^ protein blockers, which have demonstrated positive *in vitro* and *in vivo* results.

## DECLARATIONS

### Copyright

© The Author(s) 2024.
